# Factores asociados al tratamiento no exitoso para tuberculosis en pacientes previamente tratados en Cali, Colombia, en el periodo 2015-2019

**DOI:** 10.7705/biomedica.8104

**Published:** 2025-08-11

**Authors:** Julio Cesar Salvatierra Hilares

**Affiliations:** 1 Escuela Profesional de Medicina Humana, Universidad Privada San Juan Bautista, sede Chincha, Perú Universidad Privada San Juan Bautista Universidad Privada San Juan Bautista Chincha Perú

Chincha, 7 de junio de 2025

Señores

Comité Editorial

Revista *Biomédica*

Bogotá, D. C., Colombia

Señores editores:

He leído con gran interés su artículo titulado: “Factores asociados al tratamiento no exitoso para tuberculosis en pacientes previamente tratados en Cali, Colombia, en el periodo 2015-2019”. El propósito de esta carta es reconocer los aportes de este artículo y, a la vez, aportar algunas observaciones que podrían potenciar o enriquecer futuros estudios ^1^.

La investigación presenta una metodología sólida, sustentada en una base de datos extensa y minuciosa, que abarca los años 2015 al 2019. Este enfoque permite una mirada profunda a las características sociodemográficas de los pacientes y una evaluación exhaustiva de las asociaciones entre tales características y los desenlaces del tratamiento. Cabe destacar que los investigadores señalaron varios factores significativos que contribuyen al incumplimiento de la medicación, entre ellos: vivir en situación de calle, padecer enfermedades como la diabetes o coinfección con VIH, presentar farmacodependencia y abandonar el seguimiento médico ^1^. Asimismo, en un estudio realizado por Montiel et al., se identificaron factores sociales similares, como la condición de indigencia, el consumo de sustancias psicoactivas y la coinfección con VIH ^2^.

En otro estudio realizado por Caballero et al., la principal causa del abandono del tratamiento está relacionada con la atención deficiente brindada en los centros de salud y la duración prolongada del tratamiento antituberculoso. A esto se suman otros aspectos, como la carencia de recursos económicos para asistir frecuentemente a las consultas, la falta de respaldo familiar o social -ya que muchos pacientes afirmaron no contar con el apoyo de nadie-, la aparición de reacciones secundarias durante la administración de la medicación y la escasa información proporcionada sobre la terapia ^3^. Una diferencia entre las investigaciones es que el estudio realizado por Caballero et al. se relaciona directamente con la propuesta de fortalecer la atención médica y la infraestructura, mientras que el trabajo de Montiel et al. incorpora la necesidad de intervenciones dirigidas a poblaciones vulnerables, como personas farmacodependientes o aquellas en situación de calle.

También, el artículo pone sobre la mesa la relevancia de estrategias como la asesoría para la prueba de VIH, que demostró una relación positiva con el éxito del tratamiento. Este resultado es esperanzador, ya que refuerza la relevancia del asesoramiento y la educación en la adherencia al régimen terapéutico contra la tuberculosis, lo cual puede ser clave en la mejora de los resultados clínicos.

Sin embargo, considero que se podría ahondar en la exploración de las barreras específicas que enfrentan estos grupos vulnerables en el acceso y continuidad del tratamiento, particularmente aquellos en situación de calle. Por ejemplo, sería interesante explorar cómo los sistemas de salud pueden adaptar sus planes para mejorar el cumplimiento del tratamiento, como programas de acompañamiento individual, capacitación continua del personal de salud, sesiones educativas a los pacientes, implementación de programas de apoyo, mejoras en la infraestructura sanitaria y potenciación de estrategias DOTS (*Directly Observed Treatment, Short-course*), una táctica de control de la tuberculosis recomendada por la OMS ^4^.

En conclusión, como los autores mencionaron, si bien este estudio ofrece una perspectiva relevante sobre los factores de riesgo asociados al fracaso del tratamiento, la generalización de los resultados debe tomarse con pinzas, debido a la naturaleza retrospectiva del estudio. Sería importante realizar estudios prospectivos o intervenciones que puedan ofrecer una evidencia útil en tiempo real sobre la eficacia de las estrategias propuestas. Aprecio la oportunidad de haber leído y reflexionado sobre este trabajo, el cual constituye una contribución valiosa para progresar en la gestión y el control de la tuberculosis en los diferentes países.
