# Supplementary material for: Factores asociados al tratamiento no exitoso para tuberculosis en pacientes previamente tratados en Cali, Colombia, en el periodo 2015-2019
Source: Biomedica. 2025 Aug 11;45(3):473–6. [Article in Spanish] doi: 10.7705/biomedica.8104 (PMC12443420; doi:10.7705/biomedica.8104)
Supplement: Related texts

# Reply: Cartas al editor

Biomédica 2025;45:473-6

Cali, Colombia, julio de 2025

Doctor

Julio César Salvatierra Hilares

Universidad Privada San Juan Bautista

Chincha, Perú

Estimado Dr. Salvatierra:

Agradecemos sinceramente su carta al editor sobre nuestro artículo «Factores asociados al tratamiento no exitoso para tuberculosis en pacientes previamente tratados en Cali, Colombia, en el periodo 2015-2019» ^1^^,^^2^, en la cual comparte observaciones valiosas para enriquecer futuras investigaciones. Valoramos profundamente su interés y sus comentarios constructivos, en los que destaca aspectos clave de nuestro estudio y propone direcciones relevantes para avanzar en el control de la tuberculosis.

Coincidimos en la importancia de abordar las barreras para la prevención y el control de la tuberculosis, en especial las que enfrentan las comunidades vulnerables, como las personas en situación de calle o con farmacodependencia, tal como usted señala. Su sugerencia de explorar estrategias adaptadas -como programas de acompañamiento individual, comunicación del riesgo para las comunidades, capacitación continua del personal de salud y fortalecimiento de las estrategias de tratamiento directamente observado de curso corto (DOTS)- es especialmente pertinente. Estas intervenciones podrían integrarse en futuros estudios de implementación para evaluar su impacto en tiempo real y mejorar la adherencia al tratamiento.

Asimismo, apreciamos la referencia a los trabajos de Montiel *et al*. ^3^ y Caballero *et al*. ^4^, que complementan nuestros hallazgos al enfatizar la necesidad de intervenciones multifacéticas, tanto en el ámbito clínico como social. La conexión entre la calidad de la atención médica, el apoyo socioeconómico y el éxito terapéutico es un área que merece mayor profundización, tal como usted sugiere.

Con respecto a las limitaciones de nuestro estudio, coincidimos en que, dada su naturaleza retrospectiva y el análisis secundario de los datos recolectados con fines de vigilancia epidemiológica, será necesario realizar estudios adicionales que permitan la confirmación de los hallazgos. Nuestra investigación, aunada a los otros estudios referidos por usted, respalda la necesidad de priorizar las intervenciones de salud pública para fortalecer la atención primaria en salud e implementar estrategias con potencial para mitigar las barreras identificadas ^5^. Estas medidas ayudarían a mejorar la oportunidad y la calidad de la atención en las poblaciones vulnerables y a garantizar la efectividad del tratamiento.

Nuevamente, agradecemos sus reflexiones y el diálogo académico que promueve. Sus aportes enriquecen la discusión sobre cómo optimizar las intervenciones en tuberculosis, especialmente en poblaciones vulnerables. Esperamos que nuestro trabajo, junto con sus observaciones, contribuya a impulsar políticas y prácticas más efectivas en la lucha contra esta enfermedad.

Atentamente,

Liddy Varela, Jimena Ortiz, Pamela K. García, Lucy Luna, José F. Fuertes-Bucheli, Robinson Pacheco

## References

[1] Varela L, Ortiz J, García PK, Luna L, Fuertes-Bucheli JF, Pacheco R. (2023). Factores asociados al tratamiento no exitoso para tuberculosis en pacientes previamente tratados en Cali, Colombia, en el periodo 2015-2019. Biomedica.

[2] Varela L, Ortiz J, García PK, Luna L, Fuertes-Bucheli JF, Pacheco R. (2023). Fe de erratas. Factores asociados al tratamiento no exitoso para tuberculosis en pacientes previamente tratados en Cali, Colombia, en el periodo 2015-2019. Biomedica.

[3] Montiel I, Alarcón E, Aguirre S, Sequera G, Marín D. (2020). Factores asociados al resultado de tratamiento no exitoso de pacientes con tuberculosis sensible en Paraguay. Rev Panam Salud Publica.

[4] Caballero MM, Cerrufo DEJ, Cordoba MRV. (2021). Factores que inciden en el abandono del tratamiento antituberculoso en los pacientes que acuden a los subcentros de salud en la ciudad de Milagro, 2019. Más Vita.

[5] Pradipta IS, Idrus LR, Probandari A, Lestari BW, Diantini A, Alffenaar JWC (2021). Barriers and strategies to successful tuberculosis treatment in a high-burden tuberculosis setting: A qualitative study from the patient’s perspective. BMC Public Health.
